# Supplementary material for: Attenuation of the CpG island methylator phenotype and lack of WNT signalling activation restrains Kras mutant intestinal neoplasia
Source: Br J Cancer. 2026 Feb 23;134(8):1230–9. doi: 10.1038/s41416-025-03271-3 (PMC13035823; doi:10.1038/s41416-025-03271-3)
Supplement: Supplementary file 2 — Arrive checklist [file 41416_2025_3271_MOESM2_ESM.docx]

Supplementary Table 1. The total number of mice used for each outcome measure. Animals were only excluded from this study if they did not survive to their designated timepoint.

| Total number of mice, n=238 | | | |
| --- | --- | --- | --- |
|  | Histology, n=212 | DNA methylation, n=44 | RNAseq, n=19 |
| *Braf*^V637^ | 134 | 10 | 9 |
| *Kras*^G12D^ | 78 | 12 | 10 |
| WT_*Braf* | - | 10 | - |
| WT_*Kras* | - | 12 | - |

Supplementary Table S2: Sex-specific breakdown of murine serrated precursor lesions in *Kras* mutant animals

| Timepoint | Male | Female |
| --- | --- | --- |
| 10 days | 0 | 0 |
| 2 months | 0.4 | 0 |
| 5 months | 0.29 | 0.25 |
| 8 months | 1.6 | 0.88 |
| 10 months | 1.14 | 0.33 |
| 14 months | 1.83 | 0 |
| 18 months | 1 | 0.285 |
